# The use of nitrogen isotopic fractionation as a biomarker for feed conversion efficiency in pigs using blood and hair samples

**DOI:** 10.1080/10495398.2025.2473494

**Published:** 2025-03-12

**Authors:** Long Cheng, R. J. Smits, F. R. Dunshea, R. J. Dewhurst, J. J. Cottrell, S. S. Chauhan, J. Luo, H. Khanaki

**Affiliations:** a Faculty of Science, Dookie College, The University of Melbourne, Melbourne, Australia; b Rivalea Australia Pty Ltd, Albury, Australia; c Faculty of Science, Parkville Campus, The University of Melbourne, Melbourne, Australia; d Scotland’s Rural College (SRUC), Edinburgh, UK; e Sichuan Agricultural University, Chengdu, China

**Keywords:** Swine, carcass composition, proxy, breeding, isotope

## Abstract

This study aimed to 1) Establish relationships between feed conversion efficiency (FCE; live weight gain/intake) and several biomarkers in pigs using blood and hair samples, and 2) Investigate the relative FCE performance of pigs from maternal vs. terminal genetic lines fed high vs. low energy diets. 80 male pigs (Large White x Landrace) were fed for 56 days. The terminal genetic line and pigs fed the high energy diet had 5% and 15% higher FCE than the maternal genetic line and pigs fed the low energy diet, respectively. Blood nitrogen isotopic fractionation (Δ^15^N; animal δ^15^N – feed δ^15^N) explained 34% more variation of FCE, compared with the blood insulin‑like growth factor‑1 (IGF‑1). The turnover rate of δ^15^N in plasma was faster than δ^15^N in blood, suggesting that blood and plasma δ^15^N can predict long‑term versus short‑term FCE changes. Pigs fed the high energy diets showed 13% higher live weight gain, 16% greater high standard carcass weight, and 38% higher carcass backfat than those on low‑energy diets. In conclusion, Δ^15^N is a more effective FCE biomarker for pigs compared to IGF‑1. Hair samples were less predictive of FCE than blood‑derived biomarkers, suggesting further refinement in the methodology of harvesting hair samples.

## IMPLICATIONS

Using nitrogen isotopic fractionation as a biomarker for feed conversion efficiency in pigs could significantly improve the pig farming industry. Economically, it allows farmers to identify and breed more efficient pigs, reducing feed costs and improving profitability. Environmentally, better feed efficiency leads to lower feed consumption and waste, reducing the ecological footprint of pig farming. Socially, this can lead to more sustainable farming practices, promoting animal welfare by minimizing the need for live measurements. Overall, nitrogen isotopic fractionation offers a low-cost, reliable method for enhancing pig production efficiency, with broad implications for sustainable agriculture.

## Introduction

Breeding and performance assessment for feed conversion efficiency (FCE; live weight (LW) gain/intake) is a crucial productive trait for a sustainable pig industry. The FCE enhances productivity while minimizing feed costs, making it a key focus in pig breeding programs. Traditional methods to measure FCE, such as individual animal intake, are challenging to quantify in commercial production systems. Thus, utilizing biomarkers to predict FCE offers a promising alternative for performance assessment without the need for extensive live measurements.

Previous research in ruminants has identified naturally occurring nitrogen isotopic fractionation (Δ^15^N; animal δ^15^N − feed δ^15^N) as a promising biomarker for FCE. Studies in beef cattle^1^ and sheep^2^^,^^3^ demonstrated that Δ^15^N inversely correlates with FCE and nitrogen use efficiency. More efficient ruminants exhibited smaller Δ^15^N values in plasma, blood, and wool. Further investigations by Cantalapiedra-Hijar et al.^4^ indicated that liver nitrogen metabolism is a major driver of Δ^15^N, while rumen nitrogen metabolism contributed minimally to Δ^15^N.

While Δ^15^N has been validated as a low-cost biomarker in ruminants, its applicability in monogastric animals, such as pigs remains unexplored. Given the similarities in metabolic processes, there is potential for Δ^15^N to serve as an indicator of FCE in pigs. Additionally, other biomarkers, such as blood and plasma urea nitrogen^5^^,^^6^ and insulin-like growth factor-1 (IGF-1),^7^^,^^8^ have been associated with growth and metabolic efficiency in livestock. This study seeks to expand the understanding of FCE biomarkers in pigs, exploring the relationships between FCE and several biomarkers including Δ^15^N, blood and plasma urea nitrogen, and IGF-1. The hypotheses of this study are: (1) Δ^15^N in both blood and hair samples can predict FCE in pigs; (2) The δ^15^N turnover rate varies across different tissue samples and correlates with changes in FCE.

This research aims to advance the understanding of nutritional concepts and measurement techniques by establishing the viability of Δ^15^N and other biomarkers in predicting FCE in pigs, exploring the δ^15^N turnover rate in various tissues to differentiate short-term and long-term FCE changes, and evaluating the impact of genetic lines and diet energy levels on FCE performance.

## Materials and methods

The study was undertaken in strict accordance with guidelines of the Australian Standards and Guidelines—Welfare of Pigs. The procedures were approved by Rivalea (Australia) Pty Ltd Animal Ethics Committee (application No. 18P005C).

Eighty 12-week-old male pigs (Large White × Landrace (PrimeGrow™ Genetics, Corowa, NSW)) weighing 43 ± 6.3 kg (mean ± *SD*) were used in this study. Two divergent genetic (G) lines and diets containing two levels of digestible energy (DE) were evaluated using a 2 × 2 factorial design. Pigs were selected from maternal (M) *vs.* terminal (T) genetic (G) lines and fed with high (H) *vs.* low (L) energy (E) content diet. Animals were housed individually in pens in the Grower Finisher Discovery Center at Rivalea, Corowa, NSW and fresh water was offered *ad libitum*. Each treatment group had 20 pigs and they were fed individually for one week for diet adaptation followed by a measurement period. Following a period of one-week acclimation in the facility and being fed their respective grower treatment diets *ad libitum*, each pig was fed restrictively at 3 × Maintenance (Maintenance = 0.45 MJ Metabolizable Energy/kg × LW (kg)^0.75^)^9^ for an 8-week measurement period. This level of feeding was used to ensure that there were no feed refusals or wastage and that variation in feed intake did not contribute to variation in performance. The measurement period comprised week 1 to week 4 on a grower diet, and week 5 to week 8 on a finisher diet (Table 1). The pigs were sourced from the Rivalea (Australia) Pty Ltd, Corowa, Australia for this study.

**Table 1.** Feed chemical composition of diets used in the experiment.*

	High energy content grower diet	Low energy content grower diet	High energy content finisher diet	Low energy content finisher diet
Crude protein (%) (determined)	18.0	18.0	17.0	17.0
Crude fat (%) (determined)	7.3	1.9	3.8	2.3
Digestible energy (MJ/kg) (calculated)	15.5	13.0	14.5	12.2
Feed δ^15^N (‰) (determined)	1.50	2.05	2.37	2.01

The numbers are ‘as fed’ basis.

*For more details of feed formulation, please refer to Supplementary Table S1.

Total of 40 MG pigs and 40 TG pigs were selected, representing different genetic lines with either a low, or high degree of selection for growth trait, respectively. Each group was then divided into two equal sub-groups balanced for LW measured at the start of the adaptation period. One MG and TG group received HE diet, while another MG and TG group received LE diet. The diets were formulated as a pellet based on DE by the Rivalea (Australia) Pty Ltd with a target of the grower diet to contain 15.5 MJ DE/kg as-fed (HE groups) and 13.0 MJ DE/kg as-fed (LE groups), and the finisher diet to contain 14.5 MJ DE/kg as-fed (HE groups) and 12.2 MJ DE/kg as-fed (LE groups). All diets were formulated to the treatment energy levels using commercial ingredients (Supplementary Table S1).

Individual pig feed intake was quantified weekly.^10^ All pigs were weighed and measured for body length (from base of the tail to the tip of the snout) at two weekly intervals throughout the study. The FCE was calculated using the equation: FCE (kg/kg) = LW gain (kg/day) ÷ feed intake (kg/day). Feed samples from each diet type were collected before the measurement period and stored at −20 °C before chemical analysis. Blood samples were collected by venepuncture of the jugular vein from each pig on measurement days 0, 14, 28, 42, and 56. Blood was then sub-sampled into tubes to collect plasma by centrifugation at 2000 g for 5 min at 5 °C. Ten pigs per group were randomly selected to measure hair growth rate. A handful of hairs were plucked from the lower back, above the tail of each pig at the start and the end of the study. Three representative, undamaged hairs (with hair root) per pig from each sampling were selected and measured under a microscope. The hair growth rate over 56 measurement days was calculated for each group as: the average hair length at the end of the study (mm) − the average hair length at the start of the study (mm). Based on the hair growth rate for each treatment group, 80 undamaged (with hair root) hair samples collected at the end of the study were cut accordingly to exclude hair from the pre-measurement period. At the end of the study, hot standard carcass weight (Trim 1, AUSMEAT standard) and carcass backfat (P2) were measured through the commercial abattoir (Rivalea, Corowa, Australia).

Diet samples were oven dried at 65 °C to a constant weight to measure dry matter (DM) content. Diet samples were ground and scanned by calibrated near infrared spectroscopy (NIRS™ DS2500, FOSS, Denmark, located at the Rivalea, Corowa, Australia) to predict chemical composition. To remove dirt contamination, each hair sample was soaked overnight in distilled water before it was cleaned in an ultrasonic bath for 3 min. The hair was then soaked in a 0.25 M sodium hydroxide solution for 2 h before washing with distilled water. The cleaned sample was dried at 25 °C in the oven for 1 h before analysis. Dried feed and hair samples, liquid blood, and plasma samples were analyzed for δ^15^N using a Thermo Flash 2000 HT (elemental analyzer) paired to a Thermo Delta V Advantage (mass spectrometer). The analysis also quantified total nitrogen content in the plasma. Plasma urea nitrogen and blood insulin like growth factor-1 (IGF-1) were analyzed using a Cobas Integra 400 plus (Roche, Switzerland) and ELISA kit (R&D Systems, Inc, Minneapolis, MN, USA), respectively.

Statistical analysis was performed using GenStat Version 16 (VSN International Ltd, Hemel Hempstead, UK). Results were considered statistically significant at *p* < 0.05. Pig production performance, plasma, blood, and hair parameters were analyzed using two-way ANOVA, with main factors G and DE and their interaction term, based on the individual pig measurement. To examine the relationships between biomarkers and individual pig FCE, three types of regression analyses were performed: (1) Simple linear regression analysis using individual pig data, (2) Simple linear regression analysis with groups, which used individual pig data while allowed four treatment groups to be included in the model, and (3) Multiple linear regression analysis using individual pig data to investigate the use of multiple biomarker combination to predict FCE. To examine the relationships between biomarkers and treatment group FCE, simple linear regression analysis was performed using average treatment data (*n* = 4).

## Results

### Feed conversion efficiency and diet effects

The TG line and pigs fed HE diets exhibited 5 and 15% higher FCE than the MG line and pigs fed LE diets, respectively (Table 2). Simple linear regression analysis without group differentiation showed that hair, plasma, and blood Δ^15^N had negative relationships with FCE, explaining 5, 19, and 43% (*p* < 0.05) of individual pig FCE variation, respectively (Table 3). At a group level, blood and plasma Δ^15^N explained 98 and 92% (*p* < 0.05) of the between-group (*n* = 4) average variation in FCE (Table 2).

**Table 2.** Intake, live weight (LW) gain, feed conversion efficiency (FCE), carcass characteristics, biomarkers measured in blood, plasma and hair of pigs selected from maternal (M) *vs.* terminal (T) genetic (G) lines and fed with high (H) *vs.* low (L) energy (E) content diet [maternal genetic high energy (MGHE), maternal genetic low energy MGLE, terminal genetic high energy TGHE and terminal genetic low energy (TGLE)].

	MGHE	MGLE	TGHE	TGLE	*P* (G)	*P* (E)	*P* (G × E)	LSD^a^
Average 8 weeks intake (kg/day/pig)	2.39	2.41	2.33	2.40	0.42	0.28	0.53	0.078
Week 1–4 average intake (kg/day/pig) on grower diet	1.86	1.92	1.86	1.91	0.99	0.029	0.82	0.048
Week 5–8 average intake (kg/day/pig) on finisher diet	2.93	2.90	2.81	2.90	0.31	0.62	0.38	0.12
Average 8 weeks LW gain (kg/day/pig)	1.14	1.01	1.18	1.05	0.098	<0.001	0.87	0.044
Week 1–4 average LW gain (kg/day/pig) on grower diet	1.05	0.95	1.14	0.97	0.005	<0.001	0.046	0.039
Week 5–8 average LW gain (kg/day/pig) on finisher diet	1.23	1.08	1.22	1.13	0.50	<0.001	0.34	0.061
Average 8 weeks FCE (kg LW gain/kg intake)^b^	0.48	0.42	0.51	0.44	0.001	<0.001	0.27	0.014
Week 1–4 average FCE (kg LW gain/kg intake) on grower diet	0.57	0.50	0.62	0.51	0.001	<0.001	0.028	0.018
Week 5–8 average FCE (kg LW gain/kg intake) on finisher diet	0.42	0.37	0.44	0.39	0.022	<0.001	0.89	0.015
Body length change (cm/65 days)	29.1	26.7	30.3	28.2	0.11	0.008	0.83	1.65
Blood IGF-1 (ng/ml)^c^	402	334	389	331	0.62	<0.001	0.76	30.68
Plasma urea nitrogen (mmol/l)^d^	3.1	3.1	2.8	2.7	0.012	0.54	0.95	0.24
Plasma nitrogen (%)^e^	0.9	0.9	0.8	0.8	0.047	0.80	0.56	0.040
Blood Δ^15^N (‰)^f^	2.0	2.2	1.9	2.1	0.007	<0.001	0.234	0.07
Plasma Δ^15^N (‰)^g^	2.7	2.8	2.6	2.8	0.069	0.003	0.110	0.10
Hair Δ^15^N (‰)^h^	3.9	4.0	3.6	3.7	0.142	0.506	0.925	0.36
Hot standard carcass weight (kg/pig)	80.9	67.5	83.7	73.8	0.027	<0.001	0.380	4.00
Carcass P2 (mm/pig)	14.1	9.6	12.2	9.4	0.064	<0.001	0.126	1.13

^a^LSD: least significant difference *p* < 0.05.

^b^FCE = LW gain ÷ intake; measured between day 0 and 56.

^c^IGF-1: insulin like growth factor-1; average measurement of days 14, 28, 42, and 56.

^d^Average plasma urea nitrogen measurement of days 14, 28, 42, and 56.

^e^Average plasma nitrogen measurement of days 14, 28, 42, and 56.

^f^Blood Δ^15^N = blood δ^15^N (average measurement of days 28, 42, and 56) − feed δ^15^N (average measurement of grower and finisher diets).

^g^Plasma Δ^15^N = plasma δ^15^N (average measurement of days 14, 28, 42, and 56) − feed δ^15^N (average measurement of grower and finisher diets).

^h^Hair Δ^15^N = hair δ^15^N − feed δ^15^N (average measurement of grower and finisher diets).

**Table 3.** Correlations between feed conversion efficiency (FCE) and biomarkers measured in pigs selected from maternal *vs.* terminal genetic lines and fed with a high *vs.* low energy content diet.

	*R* ^2^	*SE*	*p*
Individual assessment (*n* = 80)			
Blood IGF-1^a^	0.10	0.04	0.003
Plasma urea nitrogen^b^	0.00	0.05	0.76
Plasma nitrogen^c^	0.04	0.04	0.047
Blood Δ^15^N^d^	0.43	0.03	<0.001
Plasma Δ^15^N^e^	0.19	0.04	<0.001
Hair Δ^15^N^f^	0.05	0.04	0.028
Individual assessment (*n* = 80 regression with groups)			
Blood IGF-1^a^	0.53	0.03	<0.001
Plasma urea nitrogen^b^	0.53	0.03	<0.001
Plasma nitrogen^c^	0.55	0.03	<0.001
Blood Δ^15^N^d^	0.63	0.03	<0.001
Plasma Δ^15^N^e^	0.57	0.03	<0.001
Hair Δ^15^N^f^	0.55	0.03	<0.001
Group assessment (*n* = 4)			
Blood IGF-1^a^	0.69	0.02	0.11
Plasma urea nitrogen^b^	0.00	0.05	0.95
Plasma nitrogen^c^	0.43	0.03	0.21
Blood Δ^15^N^d^	0.98	0.006	0.007
Plasma Δ^15^N^e^	0.92	0.01	0.028
Hair Δ^15^N^f^	0.10	0.04	0.37

^a^IGF-1: insulin like growth factor-1; average measurement of days 14, 28, 42, and 56.

^b^Average plasma urea nitrogen measurement of days 14, 28, 42, and 56.

^c^Average plasma nitrogen measurement of days 14, 28, 42, and 56.

^d^Blood Δ^15^N = blood δ^15^N (average measurement of days 28, 42, and 56) − feed δ^15^N (average measurement of grower and finisher diets).

^e^Plasma Δ^15^N = plasma δ^15^N (average measurement of days 14, 28, 42, and 56) − feed δ^15^N (average measurement of grower and finisher diets).

^f^Hair Δ^15^N = hair δ^15^N − feed δ^15^N (average measurement of grower and finisher diets).

### Impact of diet switch on nitrogen isotopic fractionation

Plasma δ^15^N increased by 20% on average, while blood δ^15^N increased by only 7% in response to the diet switch from the grower to the finisher diet on measurement day 28. Pigs fed HE diets also had 13% higher LW gain, 16% greater hot standard carcass weight, and 38% higher carcass P2 than those on LE diets (Table 2).

### Comparison of biomarkers to predict feed conversion efficiency

Multiple linear regression analysis indicated no improvement in FCE prediction by combining IGF-1 and Δ^15^N. Simple linear regression with groups showed that hair, plasma, and blood Δ^15^N had negative relationships with FCE. Δ^15^N measures explained more variation in FCE than traditional biomarkers (i.e., plasma urea nitrogen and blood IGF-1 explained 0 and 10% of FCE variation, respectively). The prediction showed: FCE = −0.15 × blood Δ^15^N + 0.77; *R*^2^ = 0.43; *SE* = 0.03; *p <* 0.001).

## Discussion

### Dietary influence on feed conversion efficiency

In this study, dietary treatments induced a larger difference in FCE than divergent genetic lines. This highlights that the energy content in the diet is a major driver of FCE.^11^ The difference in FCE was mainly derived from LW gain difference in this study, as feed intake remained similar across the four treatments. The higher FCE and LW gain observed for HE pigs compared with LE pigs are consistent with previous animal studies.^12–14^ Pigs fed HE diets had a 16% higher hot standard carcass weight and 38% higher carcass P2 than their counterparts fed with LE diets. The higher P2 measured in pigs offered in the HE diets may reflect the conversion of excess energy into fat stores.^15^^,^^16^ There was a trend (*p* < 0.10) for the main effect of genetic line on carcass P2. These genetic differences likely reflected the higher positive selection pressure on FCE and negative pressure on carcass P2 in the TG line genetics compared to the MG line genetics.

### Turnover rates of nitrogen isotopic fractionation in blood and plasma

The causes of the differences in turnover rates of δ^15^N in blood and plasma are mainly due to their constituent proteins. The major proteins in plasma and blood are albumin and hemoglobin, respectively, and the half-life of albumin is much shorter (∼14 days) than that of hemoglobin (∼120 days).^17^ The switch from the grower to the finisher diet on measurement day 28 provided a useful dataset to examine the relative turnover rate of the δ^15^N in plasma and blood. Similar to Cheng et al.,^2^ the current study showed that the turnover rate of δ^15^N in the plasma was likely to be faster than δ^15^N in the blood. This was supported by the evidence that across the four treatments, the plasma δ^15^N on average increased by 20%, while blood δ^15^N increased by only 7% in response to the dietary switch from a grower to a finisher diet on measurement day 28. This important finding indicates that blood and plasma δ^15^N may be used separately to predict different durations (no. of weeks or months) FCE changes in pigs. However, a longer-term study is needed to confirm this, as previous work showed it needs at least four weeks turnover time for δ^15^N to accumulate in blood and provide an adequate prediction of FCE in sheep.^2^

The Δ^15^N calculated from different average sampling date measurements for plasma δ^15^N and blood δ^15^N was explored to understand the best predictors from different time points for FCE. The analysis revealed that taking plasma δ^15^N average measurement on days 14, 28, 42, and 56, and blood δ^15^N average measurement on days 28, 42, and 56 to calculate Δ^15^N, provided the best predictions of FCE in this study.

### Relationships between nitrogen isotopic fractionation and feed conversion efficiency

As expected, simple linear regression analysis with groups showed that variation in FCE can be largely explained by the four designed treatments. However, the major finding from this study was that the simple linear regression analysis without groups showed that hair, plasma, and blood Δ^15^N had negative relationships with FCE. This result is in line with previous findings in cattle and sheep studies^1^^,^^2^^,^^18^ and is likely linked to the nitrogen metabolism process (e.g., deamination, transamination) in the pig liver.^4^ Furthermore, it is interesting to note that Δ^15^N measures explained more variation in FCE than the traditional FCE biomarkers tested in this study (i.e., plasma urea nitrogen and blood IGF-1 explained 0 and 10% of FCE variation, respectively). This is supported by the reported phenotypic correlations between IGF-1 and FCE in growing pigs ranged between 5 and 39% in an analysis, pooled data from 9 trials.^19^ Comparable to the findings of using individual pig FCE data in this study, the use of average group plasma nitrogen, hair Δ^15^N, plasma Δ^15^N, blood Δ^15^N, and plasma IGF-1 explained 43, 10, 92, 98, and 69% variation in FCE between treatment groups (*n* = 4), respectively. Low sample size (*n* = 4) tested in this study led to plasma nitrogen, blood IGF-1, and hair Δ^15^N association with FCE being not statistically significant, but the overall results indicate that plasma Δ^15^N and blood Δ^15^N have the potential to be used to differentiate four groups on FCE differences. It was clear that blood Δ^15^N was a better predictor for FCE than the hair and plasma Δ^15^N, the exact reason for this is unknown. Based on the discussion in the last section, if this result is partly due to δ^15^N turnover rate differences in animal samples, questions, such as ‘how long does it take for pig blood, hair, and plasma δ^15^N to turnover?’ ‘How would diet and genetic variations of pigs impact on δ^15^N turnover?’ should be explored in the future to unfold the truth.

### Potential use of hair nitrogen isotopic fractionation as a noninvasive biomarker

Previous work showed a significant relationship between hair Δ^15^N and plasma Δ^15^N in cattle fed low protein diet,^18^ offering the opportunity to use hair as a sample type to replace plasma samples to predict FEC. One objective of this study was to look at the potential of using a hair sample as a ‘non-invasive’ method to predict FCE. In commercial production, it would be easier for a producer to collect a hair sample and send it for analysis than rely on a qualified stockperson or veterinarian visit to take a blood or plasma sample. The findings presented in the section above highlighted the potential to use hair Δ^15^N to predict FCE, which agrees with the results from Cheng et al.,^2^ who showed a strong association between FCE and wool Δ^15^N in sheep. However, at the pig group level, hair Δ^15^N did not predict FCE well in the current study. This may be related to the site and technique used to harvest hair samples. Further, assumptions made to calculate how much hair should be cut to exclude pre-measurement period hair may have been inappropriate and caused contamination of old *vs.* new hairs from pe- and during- experimental periods, since the calculation was done according to an average growth rate measured from 10 pigs per treatment group rather than measurement from the individual pig. Future studies are needed to refine the hair sampling technique, such as shaving off a patch of hair per pig and collecting the regrowth of hair from the patch during the measurement period, which should better capture the δ^15^N signature corresponding to the pig production performance change. This technique is often used in wool growth studies in sheep.

## Conclusion

This study confirmed the potential of certain biomarkers to detect genetic and dietary differences in FCE of grower and finisher pigs. However, hair samples were not as predictive of FCE as blood-derived biomarkers. The potential of using hair as an easily harvested and noninvasive sample should be further explored with refined techniques for hair collection in pigs. The application of either a single biomarker or a combination of biomarkers for commercial estimation of FCE in a group of pigs requires further investigation.

The study demonstrated that Δ^15^N is a more effective biomarker compared to IGF-1 for monitoring FCE in different pig groups and identifying individual pigs with high FCE. The potential of Δ^15^N as a genetic biomarker for feed efficiency remains unknown, and its suitability as a genetic selection marker will depend on the heritability of the Δ^15^N trait. Further research is needed to explore these aspects and validate the use of these biomarkers in practical settings.

## Supplementary Material

pig_supp_table.docx

## Data and model availability statement

None of the data was deposited in an official repository. The data that support the study findings are available upon request.
