# Supplementary material for: The use of nitrogen isotopic fractionation as a biomarker for feed conversion efficiency in pigs using blood and hair samples
Source: Anim Biotechnol. 2025 Mar 12;36(1):2473494. doi: 10.1080/10495398.2025.2473494 (PMC12674420; doi:10.1080/10495398.2025.2473494)
Supplement: Supplementary file 1 [file LABT_A_2473494_SM4531.docx]

Supplementary Tables S1. Diet ingredients and formulation.

Raw Material		High energy grower diet	**Low energy grower diet**	High energy finisher diet	**Low energy finisher diet**
		%	%	%	%
WHEAT	[X]	72.316609	34.246666	78.510333	10.833333
BARLEY	[X]	.	25.5	.	35.465333
MILLMIX	[X]	.	20.0	.	35.0
CANOLA MEAL 37%	[X]	.	.	6.0	6.0
SOYABEANMEAL-46%	[X]	15.366667	14.266667	7.633333	8.3
MEATMEAL 60%	[X]	3.0	3.0	3.0	2.0
WATER	[X]	1.0	1.0	1.0	1.0
TALLOW-ENZYME	[X]	2.0	.	0.766667	.
TALLOW-MIXER	[X]	4.1	.	1.5	.
LIMESTONE	[X]	0.5	0.766667	0.7	0.933333
DICALPHOS BIN ADD	[X]	0.4	0.4	.	.
DL-METHIONINE	[X]	0.11	0.053333	0.03	.
VALINE H/A	[X]	0.020057	.	.	.
QUANTUM BLUE PHYTASE MICRO	[X]	0.01	0.01	0.01	0.01
ACTIGEN MICRO	[X]	0.01	0.01	0.01	0.01
LYSINE MICRO	[X]	0.546667	0.271667	0.343333	0.071667
THREONINE MICRO	[X]	0.24	0.103333	0.133333	0.013333
COPPER PROTEINATE MICRO 24%	[X]	0.041667	0.041667	0.033	0.033
TRYPTOPHAN MICRO	[X]	0.008333	.	.	.
SALT BIN MICRO	[X]	0.2	0.2	0.2	0.2
ALL SPP VIT BLEND A MICRO lar	[X]	0.025	0.025	0.025	0.025
MINERAL RUMINANT MICRO	[X]	0.031667	0.031667	0.031667	0.031667
MINERAL MONOGASTRIC MICRO	[X]	0.04	0.04	0.04	0.04
ALL SPP VIT BLEND B MICRO (abo	[X]	0.033334	0.033334	0.033334	0.033334
